# Factors affecting Dupont´s lark distribution and range regression in Spain

**DOI:** 10.1371/journal.pone.0211549

**Published:** 2019-02-15

**Authors:** Alexander García Antón, Vicente Garza, Jorge Hernández Justribó, Juan Traba

**Affiliations:** 1 Terrestrial Ecology Group (TEG-UAM), Department of Ecology, Universidad Autónoma de Madrid, C/ Darwin, Madrid, Spain; 2 C/ Vía Límite, Madrid, Spain; 3 INECO, Madrid, Spain; University of Bern, SWITZERLAND

## Abstract

In this work, we analyse factors explaining the distribution and range regression of Dupont’s lark in Spain, the only European country in which this threatened alaudid is present. Dupont’s lark is an extremely elusive and scarce species, distributed across a reduced and strongly fragmented range, showing a metapopulational structure with unknown dispersive and connective mechanisms. We used maximum entropy modelling (Maxent) on nearly 15,000 Dupont’s lark observations (1985–2015) to assess the probability of presence at a 1 km resolution across its European range. Moreover, we tested the probability of extinction by comparing pre- and post-2000 observations by means of a GLM over a subset of cells with presence-absence data. We obtained strong model fitting (AUC = 0.919), in which species occurrence was explained by low values of plant productivity (NDVI), climate (high temperature range and medium annual precipitation), land use (increasing with sclerophyllous scrubland), flat topography and human disturbance (associated with low human population density). The species also tolerates dry farming, but not other farm types or forest cover. The probability map identified two main regions known as the species' core areas: the steppes of the Iberian System and the Ebro Valley. The North Plateau is characterised by a dispersed structure of small and very fragmented patches of suitable habitat, while a succession of discontinuous probability patches form an Eastern Corridor connecting the central core areas to the southernmost populations. Finally, the model identified small and isolated patches of high probability of presence along the eastern coastline. The species tends to occur in the best available areas but, at the same time, the model revealed a large area of suitable but unoccupied habitat. Our results correct the previous estimation of occupation area from 1,480 to 1,010.78 km^2^, a reduction of 26.22%. The current distribution of Dupont’s lark is almost completely covered by Important Bird Areas (IBAs), highlighting their importance for bird conservation, but only 44.89% is included in Natura 2000 Special Protection Areas (SPAs). A comparison of pre- and post-2000 periods revealed a range contraction of 44%. Probability of extinction increased with higher temperature range and lower annual precipitation, and with decreases in population density, which suggests that this species is extremely vulnerable to both climate change and rural abandonment, due to its dependence on traditional grazing. These results suggest the need for a re-evaluation of the conservation status of Dupont’s lark in Spain. They urge the preservation of not only current extant populations, but also the unoccupied suitable areas that could be critical for metapopulation structure, and the development of policies addressing the preservation of traditional grazing.

## Introduction

The distribution of animal species is a central concept in ecology, particularly when evaluating threatened species in conservation studies, because decreases in distribution ranges are associated with increases in the risk of extinction [1]. Range reduction can promote changes in species status according to IUCN criteria [2].

Traditionally, species distribution modelling (SDM) has been based on presence and absence data, by means of generalised linear (GLM) or additive models (GAM), the latter used when nonlinear species’ responses to environmental variables are predicted. However, the use of actual absence data is a current challenge in ecology [3], not only because it is frequently missing in the study of animal species, but also because it can introduce confounding information, as areas with unconfirmed presence can indicate either unsuitable habitat for the target species or simply unoccupied locations [4]. The bias in absence data collection can be especially important when studying scarce, elusive species that are difficult to detect. Consequently, analytical methods using presence-only data have been increasingly used in recent years [5].

In this work, we use Maxent software, a presence-only procedure for modelling species distribution [6], to assess the distribution of Dupont’s lark (*Chersophilus duponti*, Vieillot 1820) in Spain, the only European country in which this species is present. Dupont’s lark is an elusive, scarce and threatened alaudid restricted to Spain, Magreb (Morocco, Algeria and Tunisia) and, rarely, to Libya and Egypt [7–10]. According to genetic analyses, the geographical origin of the species is situated in North Africa [11]. About 350,000 years ago, it diverged into two subspecies, the nominal one remaining in Morocco, Northern Algeria and Tunisia and colonising Europe, and the race *margaritae* [12] displacing to the east (Southern Algeria, Tunisia, Libya and Egypt). European and North African populations segregated during the last glaciation (24,000 years ago), and more recently several expansive processes occurred in Spain (14,000, 10,000 and 3,500 years ago) [11, 13]. Genetic flow among the Spanish and African populations is currently absent, resulting in exclusive haplotypes in the European genetic variety, although remaining as the same subspecies [11].

Dupont’s lark distribution in Spain is reduced and strongly fragmented, as it occupies a scarce habitat distributed in disperse patches in a hostile non-habitat matrix [9]. Its area of occupancy was estimated at 1,480 km^2^ [9] and divided into five main natural regions. The steppes of the Iberian Mountains (74% of the population) and the Ebro Valley (18%) are the main nuclei, followed by the North Plateau, La Mancha and Southeast regions [14, 15]. This distribution results in a metapopulational structure in which the majority of the populations have experienced a negative trend over the last years [15–18].

Dupont’s lark is an extremely habitat-selective bird, with a strong preference for natural plain steppe systems covered by low scrub (20–40 cm), minimal grass presence and a high proportion of bare ground, rejecting slopes over 10–15% [8, 19, 20–24]. It is considered principally sedentary, with observations of individuals throughout the year in the main Iberian populations [9, 25, 26].

Globally, Dupont’s lark is listed as Near Threatened by the IUCN and is included in Annex I of the Birds Directive (79/409/CEE). It is considered in Danger of Extinction in the Red Book of Birds of Spain [27] but legislation (Spanish Catalogue of Threatened Species) gives it the category of Vulnerable. The updating of its status to in Danger of Extinction in Spanish law has been suggested in response to the general negative trends in its populations [28]. The main threats to the species seem to be related to its small and fragmented distribution, which makes Dupont’s lark highly vulnerable to alterations, principally, habitat loss and fragmentation [9, 27, 29]. The last population size estimation situates the entire European population at 2,200–2,800 pairs [9], which may be currently lower considering the negative trends revealed in a recent study estimated at an average annual reduction of 3.9% during the period 2004–2015 [18]. Moreover, the Spanish population suffers from a high degree of fragmentation and isolation, with gene flow being virtually absent [30] and with a high risk of extinction in the smallest and most isolated nuclei [31].

Despite the abovementioned population decreases, no specific studies on the causes for these decreases have been carried out. Habitat loss has been mentioned as the main threat to the species due to its strong specialization in a particular type of shrub-steppe that is highly vulnerable to anthropic alteration [9]. Land use changes favoured by EU subsidies transform natural steppe-lands, both by abandonment of extensive shepherding (which results in scrub succession due to reduced grazing) or by agricultural intensification for dry farming, irrigation or reforestation (which implies ploughing of the flat scrub-covered zones occupied by the species) [27, 29]. Habitat fragmentation due to infrastructure development could also be critical for the species, as it increases distances and reduces permeability among population patches. In this sense, wind farms and linear transport infrastructures have been indicated as some of the most important factors affecting Dupont’s lark populations [27, 29]. In particular, populations in the presence of wind farms show a significantly more regressive overall trend than those in the absence of wind farms [32].

Moreover, the current global change scenario brings new concerns to Dupont’s lark distribution and conservation, given the dependence of this specialist on a very particular habitat type associated with climate attributes: strongly seasonal areas with certain levels of aridity that have poor soils with scarce and patchy vegetation development [9]. In this sense, Dupont’s lark could be highly sensitive to changes in climate, especially to those expected to produce changes in the structure and/or distribution of natural steppe-lands.

In this context, a complete and updated knowledge of factors affecting Dupont’s lark distribution in Spain is needed to evaluate: i) current occupancy range and factors determining its distribution; ii) unoccupied, but adequate habitat potentially available for the species; and iii) changes in the occupancy range and factors explaining the regression. Finally, iv) we also assess to what extent the current distribution surface is effectively included in the network of protected areas. This information on Dupont’s lark will be crucial for the management of the species and help to determine its conservation status and implement the necessary conservation measures in Spain and Europe.

## Materials and methods

### Occurrence locations

We compiled the largest georeferenced database of the species thus far, gathering 14,923 Dupont’s lark observations from various sources, both published and unpublished. The main source of data was our own dataset (Terrestrial Ecology Group–UAM), which accounted for 75% of the locations, partly published by Suárez [9]. Official information provided by the regional administrations including occurrence of the species (Andalucía, Aragón, Castilla-La Mancha, Castilla-León, Murcia, Navarra and Valencia) added 17% of the observations, and the rest (8%) came from personal observations by ornithologists and researchers. Most of the locations corresponded to the period 2000–2015 (14,476 observations; 97.0%), which includes the II National Census [9] and most of the work carried out with the species in Spain since then. The rest of the observations (447; 3.0%) belong to the pre-2000 period (1985–1999), coming fundamentally from the I National Census [19].

We used only post-2000 observations for building the species distribution model (see below), in order to maintain temporal consistency between the occurrence localities and the environmental variables available for the analysis [33] (Table 1). We used pre-2000 observations to analyse locations in which the species has gone extinct and to assess factors explaining the regression.

**Table 1:** Pool of environmental variables included in the analysis.

Variable	Description	Original data and resolution	Value in 1x1 km cell
***Topography***			
**Elevation**	Metres above sea level	DTM raster 25 m	Mean
**CV**	Coefficient of variation of the elevation	DTM raster 25 m	Cell's CV
**Slope**	Angle of incline	DTM raster 25 m	Mean
***Climate***			
**January temperature**	Mean temperature of the coldest month	Raster 1x1 km, period 1950–2000	Mean
**August temperature**	Mean temperature of the hottest month	Raster 1x1 km, period 1950–2000	Mean
**April temperature**	Mean temperature in the centre of the breeding period	Raster 1x1 km, period 1950–2000	Mean
**Temperature range**	Max. temp. of the hottest month—min. temp. of the coldest month	Raster 1x1 km, period 1950–2000	Mean
**Annual precipitation**	Annual cumulative rainfall	Raster 1x1 km, period 1950–2000	Mean
**January precipitation**	January cumulative rainfall, proxy of potential snow cover	Raster 1x1 km, period 1950–2000	Mean
**Pre-breeding precipitation**	October-to-January cumulative rainfall	Raster 1x1 km, period 1950–2000	Mean
***Land use***			
**Artificial**	Surface of CORINE 111+112+121+122+123+124+131+132+133+141+142	Vector, year 2006	Percentage
**Dry farming**	Surface of CORINE 211	Vector, year 2006	Percentage
**Other farming**	Surface of CORINE 212+213+221+222+223+231	Vector, year 2006	Percentage
**Farming+natural**	Surface of CORINE 243	Vector, year 2006	Percentage
**Farming+forest**	Surface of CORINE 241+242+244	Vector, year 2006	Percentage
**Natural grassland**	Surface of CORINE 321	Vector, year 2006	Percentage
**Sclerophyllous scrub**	Surface of CORINE 323	Vector, year 2006	Percentage
**Other scrub**	Surface of CORINE 322+324	Vector, year 2006	Percentage
**Forest**	Surface of CORINE 311+312+313	Vector, year 2006	Percentage
**Scarce vegetation**	Surface of CORINE 333	Vector, year 2006	Percentage
**Unvegetated**	Surface of CORINE 331+332+334+335	Vector, year 2006	Percentage
**Wetland**	Surface of CORINE 411+412+421+422+423+511+512, 521+522+523	Vector, year 2006	Percentage
***Vegetal productivity***			
**NDVI**	Normalised Difference Vegetation Index	Raster 250 m, two-week, 2006	Mean
**EVI**	Enhanced Vegetation Index	Raster 250 m, two-week, 2006	Mean
***Anthropic use***			
**Cattle density**	N° of sheep+goat heads/10 ha	Municipality value, 2009	Municipality value
***Disturbance***			
**Population density**	N° of inhabitants/10 ha	Municipality value, 2015	Municipality value
**Transport Network 1**	Total length of highways, motorways, national roads and high speed railways	Vector, year 2015	Sum
**Transport Network 2**	Total length of regional roads, paths and other railways	Vector, year 2015	Sum

Explanatory variables included in each 1x1 km cell of the grid. The original raster or vector data used for each predictor is indicated together with its spatial and temporal resolution, as well as how the final value of the 1x1 km cell was calculated.

### Explanatory variables

Observations were placed in a 1x1 km grid across peninsular Spain and the Balearic Islands, which results in a presence-only response variable. By means of a Geographic Information System [34], we incorporated those environmental predictors that could be relevant to the occurrence of Dupont’s lark, considering the following categories: topography, climate, land use, vegetal productivity, anthropic use and disturbance (Table 1).

In relation to topography, we included elevation and its coefficient of variation, as well as slope, considering the species’ preference for regular, flat terrains [9, 24]. These variables were extracted from the 25 m DTM provided by the National Centre of Geographic Information (https://www.cnig.es/), obtaining the mean cell’s value in the case of elevation and slope, and elevation’s coefficient of variation.

Climate data were obtained from the WorldClim database [35], with a resolution of 1 km. For each cell, as the climatic and analysis grid did not perfectly match, we extracted the mean value per cell grid for the period 1950–2000 of the following variables: mean temperature in January, August and April (meaning thermal stress in the coldest, hottest and mid-breeding periods); temperature range (difference between the maximum temperature of the hottest month and the minimum temperature of the coldest one, to account for the degree of seasonality); annual precipitation, associated with primary productivity [36, 37], and assumed to be a useful indicator of food availability [38, 39]; January precipitation (as a proxy of potential snow cover in winter); and pre-breeding precipitation, calculated as the cumulative rainfall from October to January, both included, to account for influence of food availability during the non-breeding period. For a similar approach see [40].

Land use information was obtained from the CORINE Land Cover program of the European Environment Agency (https://www.eea.europa.eu/data-and-maps/data). We used data from CORINE 2006 to maintain temporal consistency with the pool of observations used for Maxent analysis (2000–2015). We grouped original categories into other synthetic ones of ecological meaning according to Dupont’s lark habitat use (Table 1), calculating the percentage of each 1x1 km grid cell occupied by the following categories: artificial, dry farming, other farming, farming+natural mosaic, farming+forest mosaic, natural grassland, sclerophyllous scrub, other scrub, forest, scarce vegetation, unvegetated, and wetland (Table 1).

Primary productivity was estimated by means of two vegetation indices, NDVI and EVI, provided by the University of Natural Resources and Life Sciences of Vienna (http://ivfl-info.boku.ac.at/index.php/eo-data-processing/dataprocess-global). For each 1x1 km cell, we calculated the mean value of year 2006, from two-week data at a resolution of 250 m.

For the anthropic use factors, we considered the presence of sheep and goat cattle as an important variable influencing habitat structure and food availability for the species [9, 15]. We calculated the value of sheep+goat density (heads/10 ha) in 2009 at a municipality level from the National Institute of Statistics database (http://www.ine.es/), assigning to each grid cell the value of the municipality to which it belongs, calculating the area-weighted mean when more than one municipality intersected with the same grid cell.

Finally, we added three variables related to human disturbance. We estimated population density (inhabitants/10 ha) in each 1x1 km grid cell using municipality data with the same method and source as for cattle density. The effect of transport network was incorporated with two different predictors (Table 1), obtained from the National Centre of Geographic Information: total length of highways, motorways, national roads and high speed railways inside each grid cell (transport network 1), and that of regional roads, paths and other railways (transport network 2).

Previous to running the probability model, we evaluated the correlation among the explanatory variables to avoid redundant information and misleading interpretation of the variables' contribution to the probability model [41]. We used Pearson’s correlation coefficient and discarded one variable of each pair when pairwise r>0.75 (S1 Table) using expert criteria to maintain those variables that offer a better interpretation of the model for applied management and conservation of this particular species.

### Probability model

The maximum entropy modelling algorithm was used to model the potential distribution of Dupont’s lark in Spain. We used Maxent software [6] to obtain a presence probability value for each 1x1 km cell of the grid. Maxent is a machine-learning method based on the principle of maximum entropy and has been employed widely in many ecological studies (for further details see [4, 42]). Maxent models have been shown to yield one of the highest quality predictions among several modelling methods and the best performance for low sample sizes [43].

To deal with the potential bias due to unequal sampling effort across the study area, we evaluated two typical correction methods: occurrence locations bias and optimal background surface [44–47]. We selected the best fit model by means of the ROC curve and AUC value [48]. To do so, we ran the model with all explanatory variables (after removing the autocorrelations; S1 Table) in the different scenarios resulting from all possible combinations of both correction methods. To control for occurrence locations bias, we evaluated two alternative observation files, one comprising the whole set of post-2000 georeferenced locations (14,476) and the bias-corrected one, using only one observation for each occupied cell (1,370), situated in the cell’s centroid [49]. On the other hand, we assessed different background scenarios, buffering 10, 25, 50 and 100 km around the occurrence cells, as well as the all-area alternative [47]. We obtained 10 different models from which we selected the alternative that offered the highest AUC value in our case study.

Using the model with the most suitable bias correction, we selected those variables that accounted for 95% of the contribution and discarded the rest. We ran the model with the selected predictors, with 15 iterations, obtaining an averaged result. Additionally, we included a bias file considering the unequal geographical sampling effort along the study period, using as an estimator the number of sampling days at the province level. A regularization multiplier value of 2 was used to obtain smoother curves.

Model fitting was evaluated by means of the ROC curve and the AUC value, and the importance of each environmental variable was determined by an inclusion-exclusion jackknife process, which allows the scoring of the predictive capacity of each variable itself, as well as fit lost when removing that predictor from the model.

### Probability of presence and potential distribution maps

Based on Maxent results, we built a Dupont’s lark probability of presence (habitat suitability) map at 1x1 km resolution. We built the potential distribution map by assigning categorical classes to the probability value. Thus, we assigned to each unoccupied cell the category of potential or improbable presence of the species. We used as a threshold the average value of probability of all cells with confirmed presence during the study period, following the average suitability approach [50], considering that this mean value represents the typical environmental conditions selected by Dupont’s lark. Thus, we obtained for each cell the category of confirmed, potential (over the threshold) or improbable presence (under the threshold).

### Estimation of occupancy and potential surface

To avoid the risk of overestimating the areas of occupancy and potential occurrence as calculated by simply the sum of cells in the grid, as high habitat heterogeneity can be found within a 1x1 km surface, we clipped the resulting cells with habitat and slope layers. In the first case, we built a vector layer including the most reliable habitat where the species occurs by intersecting all post-2000 observations with the CORINE 2006 layer (for presence locations and environmental variables to match temporarily), and selected those categories that accounted for 95% of the observations. After choosing those preferred land use categories, we built a layer representing the current distribution of the preferred habitat by selecting them from the CORINE 2012 data, as the most recent land use information available. We then clipped the 1x1 km cells with this habitat layer. In the case of slope, we clipped out the surface over 15%, which is the top limit of the species’ tolerance [19, 21, 24]. With both habitat and slope corrections we obtained a more accurate estimation of occupied and potential surfaces for the species.

### Management and protection

We evaluated whether the current distribution of Dupont’s lark has been effectively identified as important for conservation, and to what extent the regional Administrations have included the species' distribution in the network of protected areas.

First, we intersected the layer of IBAs (Important Bird Areas Programme of BirdLife International, updated to 2010) to detect the number of presence cells that are included in the Spanish IBAs network. These areas are selected through quantitative ornithological criteria that ensure that the selected sites have true significance for the conservation of bird populations, but they do not imply statutory protection. To evaluate the extent of Dupont’s lark habitat that is truly protected we used the layer of Special Protection Areas (SPAs, updated to 2016) based on the Birds Directive 2009/147/EC, which represent areas with specific management for bird conservation. We avoided the use of other, more general protection categories (Regional, Natural or National Parks) that could be confusing, as their broad conservation aims may lead to implementing policies that are not necessarily positive for birds or, in this particular case, for the Dupont’s lark, such as land reforestation [27, 29]. Finally, we quantified the distribution surface included in IBAs and SPAs, and compiled the list of sites affected by Dupont’s lark presence.

### Factors explaining range contraction

We compared changes in the cells occupied in pre- and post-2000 periods to estimate a trend in the distribution area and to explore factors that could explain changes between the two periods. As the II National Census (post-2000 observations [9]) involved a stronger sampling effort than the first (pre-2000 observations [19]), we only evaluated the local extinction process occurring in the 320 cells occupied in the I National Census, and later resampled during 2000–2015. This data set allowed us to carry out a presence-absence analysis (see below).

Firstly, we considered habitat changes as a potential factor for a cell to be extinct. We evaluated the change between the two periods for the following variables: adequate habitat (sclerophyllous scrub), suboptimal (natural grassland and spaces with scarce vegetation), intensification (dry farming, irrigated land, vineyard, fruit and olive grove, other crops), reforestation (coniferous forest) and abandonment (broad-leaved forest and woodland-shrub), which we built from the original CORINE vector layers. To obtain a value of the intensity of change for each variable we calculated the difference between the percentages occupied in 2012 and 1990 in each cell, according to both available CORINE datasets. In the same way, we calculated the difference in the NDVI annual mean between the two dates (1999–2015) to account for changes in primary productivity.

To account for changes in anthropic effect, we estimated changes in sheep+goat density using data from the Spanish Institute of Statistics database (1999–2009). We calculated the difference in density of sheep+goat (heads/10 ha) between the two years, and assigned a value by using a weighted mean as explained above. We did the same to extract a population density change for each cell (inhabitants/10 ha), using available data from the same source (years 1996 and 2015).

We obtained climate data for the series 1993–2011 for annual precipitation and temperature annual range, from the National Agency of Meteorology. Using those stations with at least 5 years of data, one year previous to 2000 and one posterior to 2006, we obtained data from 2,546 stations for annual precipitation, and from 1,391 for temperature range. For each station, we calculated the interannual trend by means of the slope of a linear regression. We interpolated the values of the slope for all the stations to assign a trend value (slope) of temperature and range to each cell. We used kriging interpolation [34], a geostatistical method that includes autocorrelation and weights the surrounding measured points to predict a value for each grid cell [46].

Finally, and due to the negative effect attributed to wind farms on population trends of this species [32], we recorded the presence of wind turbines in the 320 cells at three different distances from each pre-2000 occupied cell: within the 1x1 km cell, and in buffers of 2 and 4 km, the last being the maximum distance at which their effect on Dupont’s lark populations has been reported [32]. We used updated PNOA orthophotographs (2014–2015) from the National Centre of Geographic Information to geolocate each wind turbine in GIS software.

We ran a logistic regression model to evaluate the probability of extinction according to the set of predictors. As a response variable, we used 0 (extant) for those cells with post-2010 Dupont’s lark presence and 1 (extinct) for those cells with no post-2010 presence. We used post-2010 presences to adequately evaluate the effect of wind farms, which were mainly installed in Spain during the years 2008–2010 due to the national regulations introduced by the Spanish Renewable Energy Plan 2005–2010. We used model averaging to evaluate the effects, building all possible combinations of variables by means of the *dredge* function in the *MuMIn* library in R [51], and selecting the plausible models following Akaike’s Information Criterion (using the 95% confidence set). We averaged the selected set of models to obtain for each explanatory variable a weighted estimator, the unconditional standard error [52], z and P values, which allowed us to identify statistically significant effects.

## Results

### Explanatory variables

After the correlation analysis, the original 28 variables were reduced to 23. Four climatic variables and one vegetation index were removed. Among the rainfall variables, we removed both January and pre-breeding precipitation while maintaining annual precipitation, which we considered to be a more useful proxy for food availability. From the thermal predictors, we preserved August mean temperature and temperature range. Finally, as both vegetation indexes were highly correlated, we kept NDVI and removed EVI, as the latter works specifically well in high biomass areas [53].

### Probability model

The correction method that offered the best fit model (AUC = 0.926) was the use of the unbiased occurrence locations file, maintaining only one observation of each occupied cell, and the complete surface a as background extension (S2 Table). Eleven variables accounted for 95% of the contribution and were used to build the final averaged model, which included plant productivity (NDVI), land use (forest, other farming, dry farming, sclerophyllous scrub and farming+forest), climate (temperature range, annual precipitation and August temperature), disturbance (population density) and topography (slope).

The averaged model showed a good fit, with an AUC of 0.919±0.006. NDVI accounted for the highest contribution (19.90%), followed by habitat and climatic predictors. Slope contributed the least (Table 2).

**Table 2:** Variables included in the averaged model.

Variable	Contribution %	Cum. %
**NDVI**	19.90	19.90
**Forest**	13.60	33.50
**Other farming**	11.10	44.60
**Dry farming**	10.90	55.50
**Temperature range**	10.80	66.30
**Annual precipitation**	7.90	74.20
**Sclerophyllous scrub**	6.80	81
**Farming+forest**	6.20	87.20
**Population density**	4.80	92
**August temperature**	4.70	96.70
**Slope**	3.40	100.10

Variables included in the final averaged model, after removing autocorrelations and selecting those that accounted for 95% of the contribution in the first run of the model. Variable contribution and cumulative contribution are indicated in each case.

**Fig 1:**
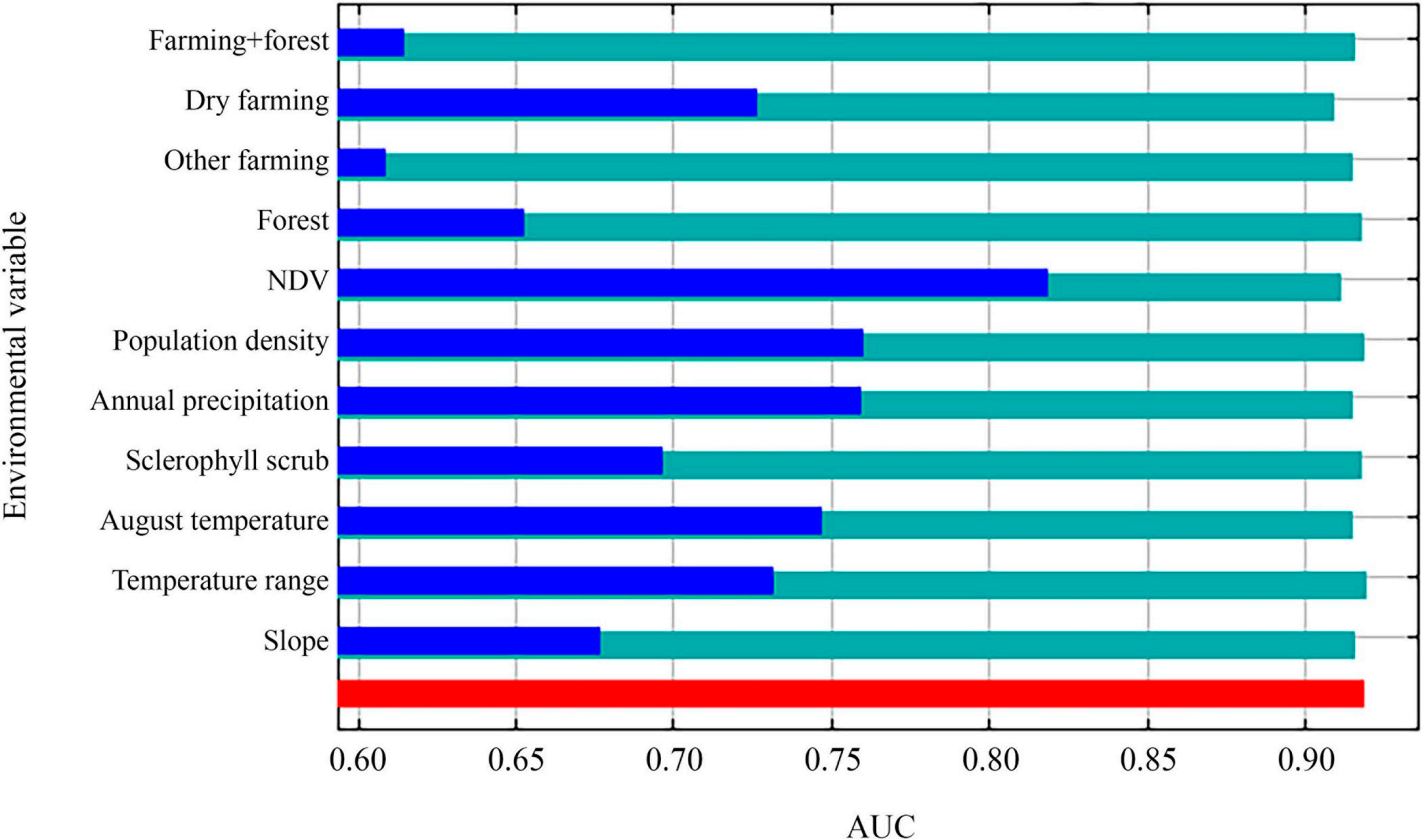
Variables importance. Jackknife test showing the importance of each variable independently, when building the Maxent model excluding the rest of the predictors. Importance estimated by means of the AUC value.

The jackknife test to evaluate the independent importance of each variable identified three with a high predictive power (AUC>0.75): NDVI, population density and annual precipitation (Fig 1).

**Fig 2:**
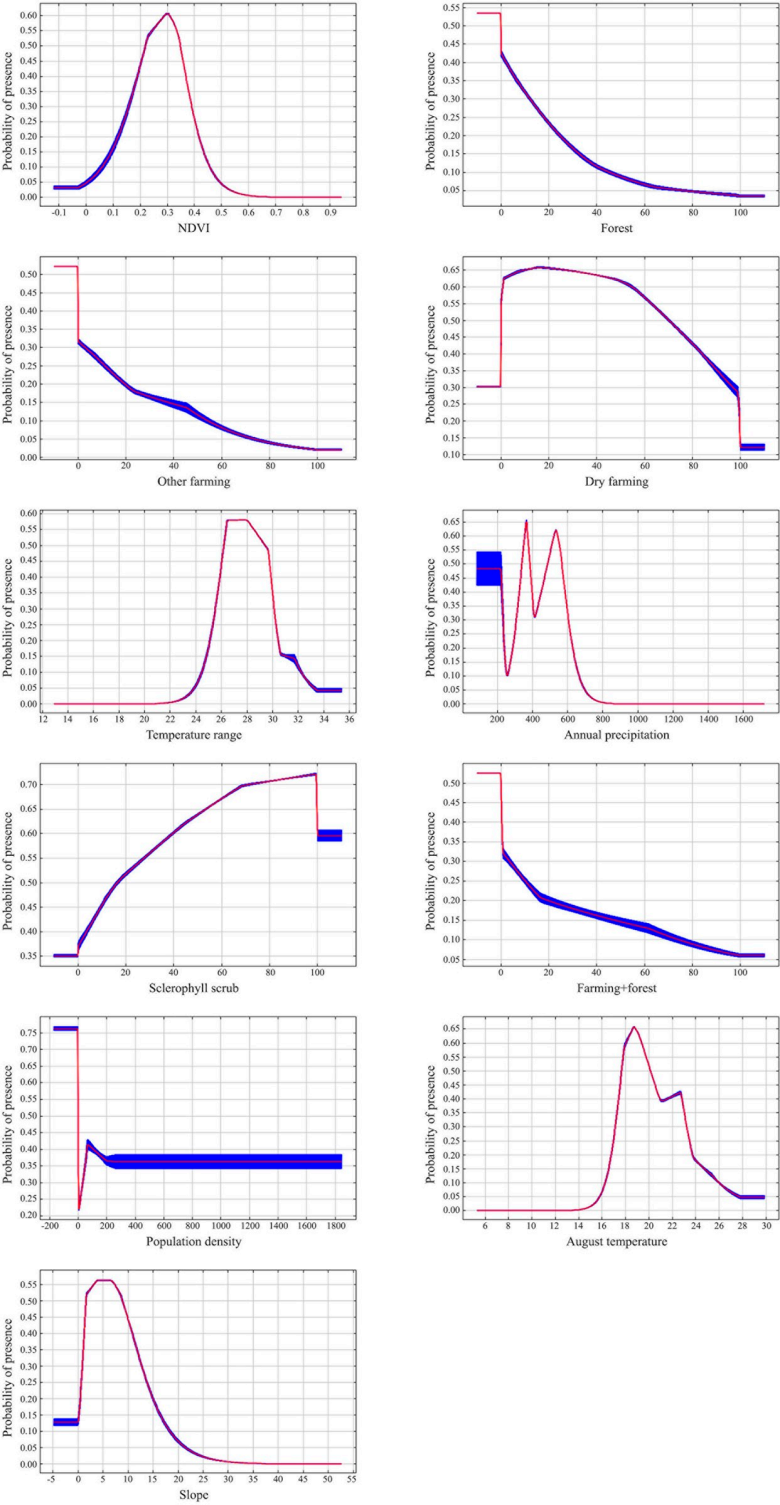
Variables effect. Effect of the 11 environmental variables included in the final model of Dupont’s lark probability of presence. Each curve represents a different Maxent model created using only the corresponding variable. The curves (red line) show the mean response of 15 Maxent iterations and the blue area is the mean +/- one standard deviation. A regularization multiplier of 2 was used in Maxent to obtain smoother curves.

Climate predictors showed a strong preference of Dupont’s lark for continental areas with a high seasonality (temperature range of 26–30 degrees), hot summers (mean August temperatures around 20 degrees) and medium annual precipitation (probability of presence decreases dramatically when annual rainfall is over 600 mm) (Fig 2).

NDVI showed an optimum around low-medium values (0.2–0.4, corresponding generally to scrubs and grasslands), where the probability of presence reached around 55–60%. Below NDVI values of 0.1, which usually corresponds to a lack of vegetation, desert/sub-desert or areas covered by rock or snow, Dupont’s lark probability of presence was around 10–15%. The probability of presence decreased progressively with increasing NDVI values, corresponding to denser vegetated areas and reaching levels close to 0 at NDVI values > 0.6 (Fig 2).

Land use predictors included five categories: percentage of sclerophyllous scrub in the grid cell had a positive effect on the probability of presence, while forest and different farming uses affected presence differently. Thus, the preference of presence of the species increases with dry farming until it reaches 50% of the grid cell surface, descending subsequently to values close to 10% of probability of presence when the cell is totally covered by dry farming (Fig 2). For other farming uses (irrigated land, rice fields, vineyards, fruit trees, olive groves and pastures with high human impact), the species showed a lower tolerance, with a more rapid decline and probability values close to 0% when this land use entirely occupied the cell (Fig 2). The same response was found for the farming+forest variable. The coverage of forest use generates a rapid decrease in the probability of presence in the cell, reaching values under 10% when woodland occupies half of the surface area.

According to topography, Dupont’s lark selected flat surfaces, with slopes showing an optimum around 5% and a rapid decline to values of 15–20%, when probability of presence is around 5–10% (Fig 2).

Finally, low population density produced a slight increase in Dupont’s lark probability of presence (over 40%), stabilised at lower values afterwards (Fig 2).

### Probability of presence and potential distribution maps

**Fig 3:**
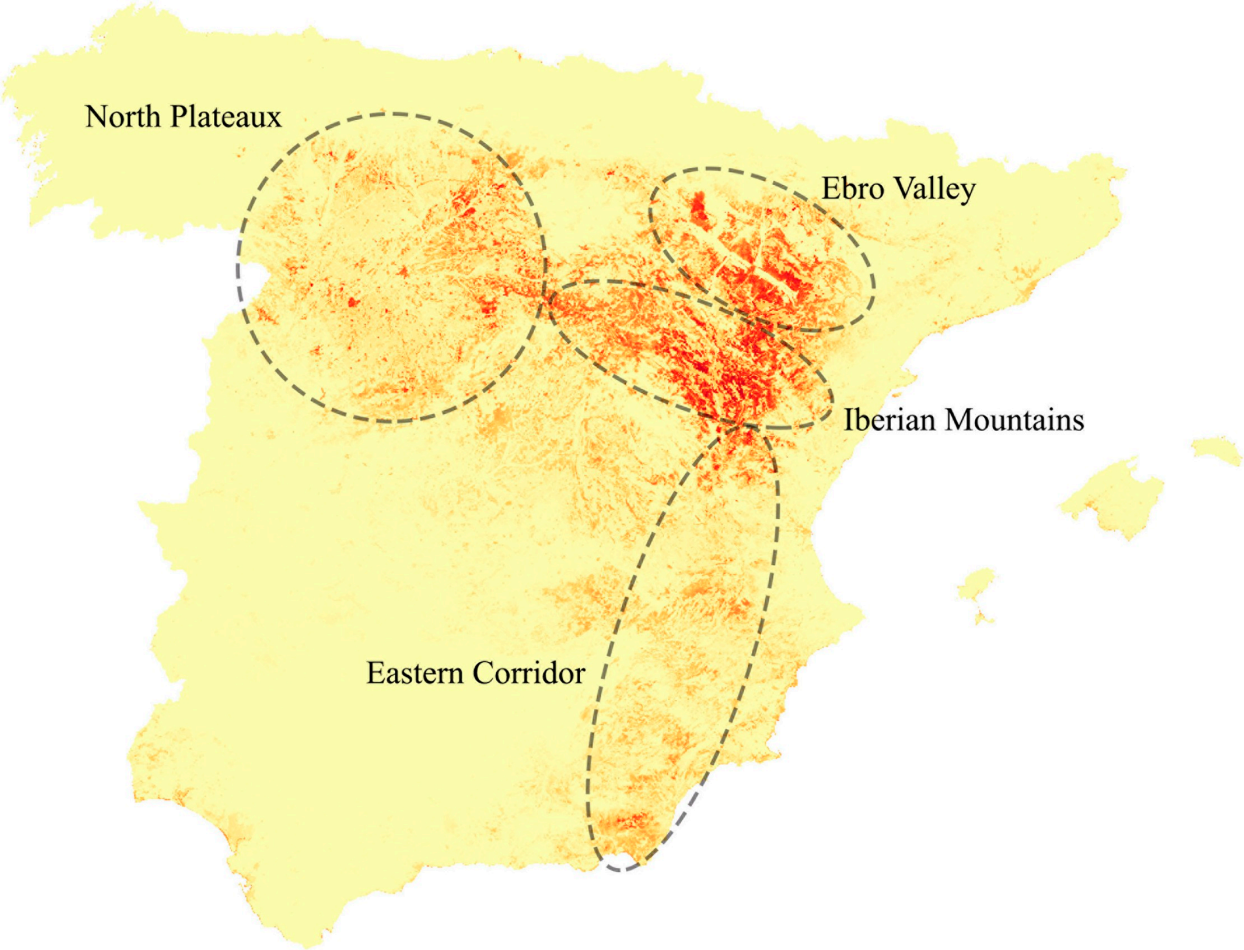
Map of probability of presence of Dupont’s lark in Spain. The colour range represents the lowest probabilities in yellow and the highest values of probability (0.82) in more intense tones of red. Four main areas are defined by the highest values: two large and continuous areas in the Iberian Mountains and the Ebro Valley, a strongly fragmented area in the North Plateau and an Eastern Corridor, with lower values of probability of presence, connecting the core areas with the southernmost range of the distribution. Finally, several coastal areas show high values of probability, although they are dispersed in small and isolated patches.

The probability of presence (habitat suitability) map (Fig 3) showed two main large and continuous areas in the Iberian Mountains and the Ebro Valley with the highest probability of presence values. A strongly fragmented distribution is shown in the North Plateau, with several but small high probability areas included in a low-to-medium probability matrix. A group of small-to-medium size patches of high probability of presence seems to form an Eastern Corridor connecting the core areas of the distribution with the southernmost populations of the species. Finally, the model revealed small and isolated patches of high probability of presence along the eastern coastline, especially in the Mediterranean and southwest regions.

1,370 cells had confirmed presence during the post-2000 period, of which the mean probability value was 0.49 (min. 0.004, max. 0.82). For the rest of the study area (500,606 cells), the mean probability of presence was 0.05 (min. 0.00, max. 0.81, revealing the existence of cells with very high probability values that are apparently unoccupied). Within the cells with a confirmed presence, 768 (56.06% of total) had a probability higher than the mean, and 602 (43.94%) had values under this threshold.

**Fig 4:**
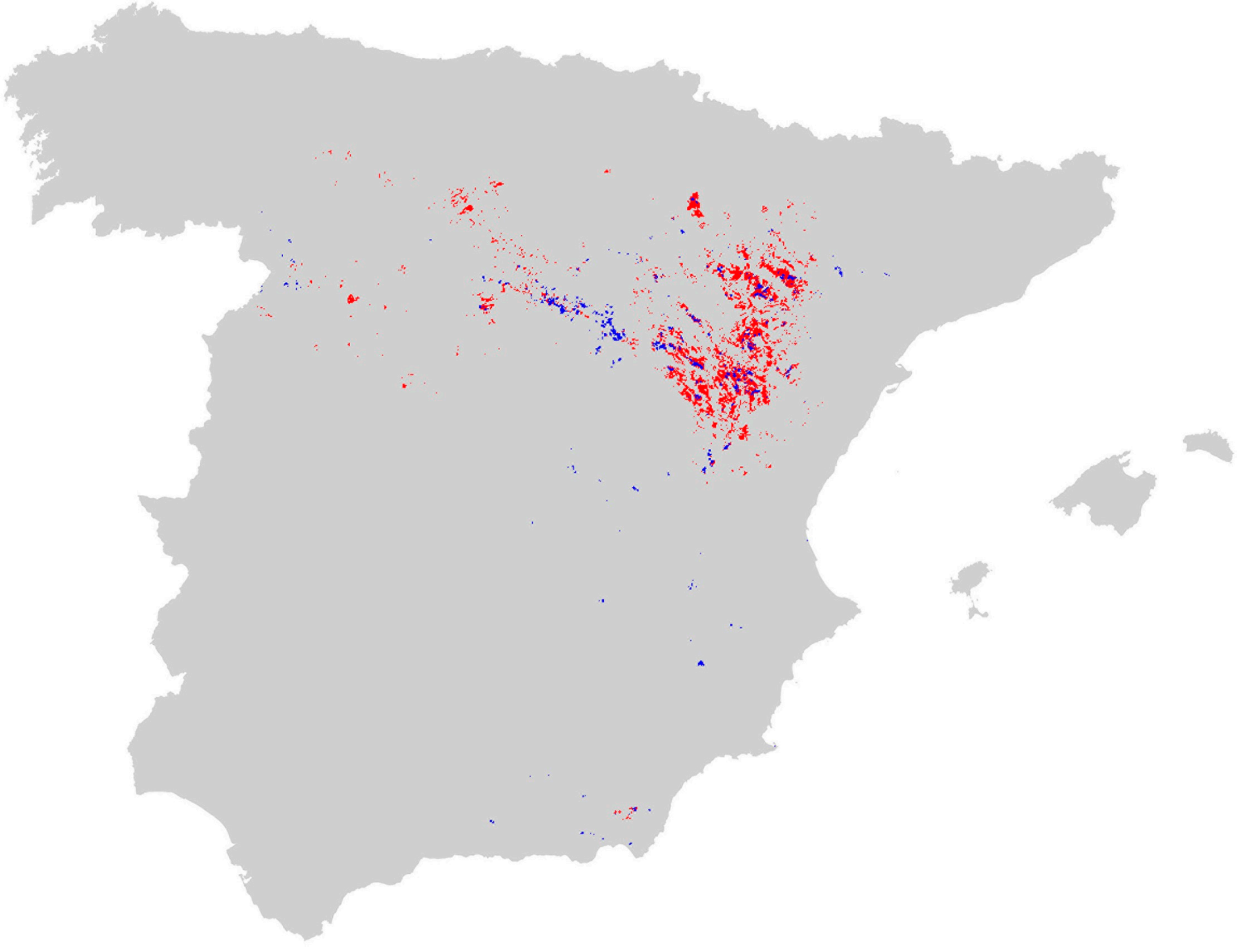
Map of potential distribution of Dupont’s lark in Spain. Grey indicates improbable presence (<0.49 probability threshold); blue, potential but unconfirmed presence (>threshold, 5,575 cells); and red, confirmed presence (1,370 cells). The map in SHP format is provided in Supporting Information (S1 File).

The species showed a trend toward occupying the best available areas (Table 3). However, the model identified a high number of cells (5,575) with no confirmed presence but with a probability value higher than the mean of the actual presence locations. As a result, the potential distribution map (Fig 4 and S1 File) suggested a relevant area of suitable habitat available for Dupont’s lark, with unknown presence.

**Table 3:** Frequency distribution of occurrence cells.

Probability of presence	N° cells	Percentage
**0–0.1**	60	4,38
**0.1–0.2**	71	5,18
**0.2–0.3**	105	7,66
**0.3–0.4**	157	11,46
**0.4–0.5**	230	16,79
**0.5–0.6**	266	19,42
**0.6–0.7**	330	24,09
**0.7–0.8**	151	11,02
**Total**	1370	100,00

Distribution of the 1,370 cells with confirmed presence in 10% step ranges of probability of presence. Dupont’s lark tends to occur in the cells with higher values of probability.

### Estimation of occupancy and potential surface

Ninety-five percent of Dupont’s lark post-2000 observations occurred in five CORINE categories (S3 Table): Sclerohyllous scrub (code 323), natural grassland (321), scarce vegetation (333), farming+natural mosaic (243) and dry farming (211), and were interpreted as the preferred habitat of the species. They were selected from CORINE 2012 and dissolved to create the habitat layer to clip the obtained 1x1 km cells.

After clipping the 5,575 cells of potential distribution with the habitat and slope correction layers, the surface was reduced to a corrected estimation of 2,639.49 km^2^, a decrease of 52.65%. Moreover, considering that a minimum patch size of 20 ha is necessary to maintain Dupont’s lark presence [9, 54], we removed all single polygons smaller than this threshold and obtained a more accurate potential distribution surface area of 2,393.43 km^2^. Following the same rule, as the species requires no less than 200 ha of continuous habitat to settle a viable population [54], the potential breeding surface would be 1,670.79 km^2^.

In the case of the 1,370 cells with confirmed presence, after the correction, the area of occupancy was reduced by 26.22%, from 1,370 km^2^ to a corrected value of 1,010.78 km^2^. Following the same previous criteria, the most probable surface of occupation when removing the patches smaller than 20 ha was 965.45 km^2^, and the area adequate for settling breeding populations was 697.91 km^2^.

### Management and protection

Sixty-seven IBAs intersected with occupied grid cells of Dupont’s lark in Spain. The top ranked in importance, according to the surface occupied by the species is IBA n° 81 (‘Páramos de Layna y Medinaceli’), with 118.70 km^2^ (S4 Table). Thirty-eight SPAs intersected with occupied grid cells of Dupont’s lark, with SPA n° ES0000302 (‘Parameras de Blancas’) having the largest surface area occupied by the species at 70.77 km^2^ (S5 Table). The current distribution of Dupont’s lark in Spain is extensively covered by IBAs (94.53%), but only minimally covered by the SPA network (44.67%) (Table 4).

**Table 4:** Occupied surface with recommended and effective protection.

	N° of cells	% of total occupied surface
**In IBA**	1295	94.53
**In IBA and SPA**	612	44.67
**In IBA but not SPA**	683	49.85
**Only in SPA**	3	0.22
**Total**	1370	100.00

Number of grid cells and percentage of the total surface covered by IBAs (Important Bird Areas: recommended protection) and SPAs (Special Protection Areas: specific protection measures for birds, Natura 2000 Network).

### Factors explaining range contraction

One hundred forty-one out of the 320 cells with confirmed presence during the pre-2000 period had no presence in the post-2000 series and were considered extinct, which results in a contraction of 44.06% of the occupation.

The averaged model showed that cell extinction was associated with a reduction in population density and annual precipitation, and with an increase in temperature range (Table 5).

**Table 5:** Averaged model.

Variable	Estimate	Importance	USE	z value	P value	
**(Intercept)**	-1.168	1	0.195	5.985	< 0.001	
**Population density**	-0.826	1	0.294	2.801	0.005	*
**Annual precipitation**	-0.698	1	0.178	3.922	< 0.001	*
**Temperature range**	5.535	1	1.541	3.591	< 0.001	*
**Wind farms in 2 km**	-0.967	0.70	0.920	1.051	0.293	
**Wind farms in 4 km**	0.365	0.56	0.455	0.802	0.422	
**Cattle density**	0.105	0.52	0.142	0.738	0.460	
**Reforestation**	0.073	0.45	0.119	0.614	0.539	
**Adequate habitat**	0.042	0.34	0.108	0.394	0.693	
**Intensification**	0.013	0.27	0.070	0.193	0.846	
**Suboptimal**	-0.002	0.27	0.089	0.023	0.981	
**Wind farms within cell**	-0.017	0.28	0.729	0.024	0.980	
**NDVI**	-0.007	0.26	0.076	0.101	0.919	
**Abandonment**	0.010	0.27	0.075	0.134	0.893	

* Significant effect.

Results of the averaged model to evaluate the probability of extinction of Dupont’s lark between pre-2000 and post-2000 periods. Cell extinction was associated with a reduction in population density (rural abandonment) and annual precipitation, and with an increase in temperature range.

## Discussion

Our model of probability of presence for the European range of Dupont’s lark offered a very strong fit and is generally in agreement with the previously known distribution [9]. The global distribution of the species is mainly explained by climatic factors and vegetation index, with less contribution of habitat descriptors. The effect of land uses and slope on species presence is consistent with habitat selection studies [8, 19–24]. Although the species tends to occur in the best available areas, we have found a large surface area of unoccupied habitat (or at least, with unconfirmed presence) with a high probability of presence, which suggests the possibility of finding new occupation areas if appropriate censuses are conducted. The map of probability of presence shows a strongly fragmented scenario, with areas of a high probability value immersed in a matrix of medium or low probabilities. Nearly half of the currently occupied distribution is excluded from the SPA Natura 2000 network. The trend in the distribution range shows a dramatic situation, with a contraction of almost half of the range in the last 25–30 years. The probability of cell extinction increased with decreases in population density, which could be linked to the abandonment of traditional extensive grazing linked to rural depopulation. In addition, an increase in thermal extremes and aridity were associated with a higher probability of extinction, of particular concern given future global change scenarios.

Dupont’s lark distribution in Spain is well known since the II National Census [9] and our results of the probability model agree with the general description of the main large-scale geographical units, revealing no unknown nuclei of high probability of presence. The Iberian Mountains and the Ebro Valley are the main core areas, as they represent the typical habitat of the species [8, 19, 21–24] in the largest and most continuous extension found in Spain. The rest of the distribution (North Plateau, La Mancha and the Eastern Corridor) shows a high degree of isolation and fragmentation of adequate habitat immersed in a territory matrix of low probability of presence. According to genetic studies, these strongly fragmented areas are exposed to a high risk of extinction due to their isolation and small patch size [30, 31].

This degree of fragmentation has been described as the main threat to Dupont’s lark, because it makes the species strongly vulnerable to alterations, mainly habitat loss [27, 29]. In this sense, measures to protect natural steppe lands from abandonment and intensification are necessary, both in the known breeding nuclei and in the smaller and more isolated areas of high probability of presence that could be critical to the whole metapopulation structure. Research on metapopulation connectivity and the role that the unoccupied-but-adequate habitat patches may play in the whole structure is needed to determine management priorities and the most important areas for preservation.

While the distribution range obtained by the probability model is similar to previous information, the quantification of the occupied surface is notably different. We correct previous estimations of 1,480 km^2^ [9] by -26.22%, thus obtaining a final occupation surface area of 1,010.78 km^2^ after the habitat and slope cut-off criteria. We find this result to be more accurate and reliable because the clear rejection by Dupont’s lark of unsuitable habitats and high slope areas [9, 24]. Indeed, we believe that our cut-off criteria avoid overestimations of the occupied surface area. This result must be taken into account to update the threat status of the species in Europe and Spain. Moreover, our correction is rather conservative, because of the criteria followed to build the cut layers. The habitat layer included five categories to account for over 95% of the observations, but three of these categories only account for 2,290 observations, while the other two account for 11,961 (82.72%). If the habitat correction layer had only included the first two categories, the resulting area of occupancy would have been even smaller. In the same way, the slope layer was built with a threshold of 15%, but both our model and previous studies show that species avoidance starts at slopes of 10% [8, 9, 19, 21–24]. In any case, the actual occupied surface area by Dupont’s lark in Spain indicates a concerning trend.

In regards to the potential distribution area, the decrease after habitat and slope corrections was 52.65%, which indicates that the adequate habitat within the potential distribution cells is more fragmented than in the presence-only ones. This could help explain the high number of potential habitat cells unoccupied by the species. The model could be assigning high scores of probability of presence to cells that, while including some proportion of optimal habitat, also contain a high portion of inadequate habitat, and the species may reject these more fragmented cells in favour of those with a more continuous adequate surface [54]. Although we have partially corrected this effect by discarding those individual patches smaller than 20 ha, our method may fail to detect the effect of the patch’s shape, which constrain its occupation by the species. Dupont’s lark may prefer not only continuous, but also compact-shaped, patches to other ones that, despite being larger than the minimum surface area required, present a more irregular shape. More research is needed on this topic.

Nonetheless, after the correction, we obtained a large surface area of potential adequate habitat (2,393.43 km^2^) compared to the occupied area (1,010.78 km^2^). We suggest several complementary explanations for this discordance. First, this could be a result of an insufficient sampling effort that may have failed to detect the species in cells where it was actually present during the post-2000 period, meaning that part of these cells would be false-absences to some extent. For instance, a specific survey carried out during 2017 in 49 high probability but to date unknown localities in Central Spain using a new technology with Automatic Recording Units revealed eight previously unrecorded occupied patches, with a total of 34 males [55].

These last results suggest that our model may be working properly. However, there could still be a large proportion of potential, but apparently unoccupied, habitat. If the species were truly absent from those cells, we should consider model inaccuracy, and the possibility that we modelled habitat distribution better than the species distribution. Additionally, although the model worked well (given the high AUC value obtained), we also should consider the possibility of constraining forces acting at the same time, making some cells unavailable despite the high probability values, for example, due to the patch’s shape as mentioned above. Finally, the absence of Dupont’s lark in a high number of cells with adequate habitat may reveal the incapacity of the species to colonise them (for example, due to a low emigration rate from breeding areas or to a lack of connectivity among these unoccupied habitats) or just a temporal absence during the sampling year following a typical metapopulation extinction-colonization process [56–58]. In this sense, we view such habitat availability as an opportunity for species management, as it may be crucial for metapopulation structure and dispersive processes, gene flow and species conservation. Thus, species management plans should take into account the preservation and monitoring of these areas. Finally, the areas of suitable habitat that still account for more than 200 ha of continuous surface (1,670.79 km^2^) must be considered of maximum and urgent conservation priority, with an aim toward preventing their reduction or fragmentation due to land use changes. This is especially relevant when considering that just 615 out of 1370 (44.89%) cells with Dupont’s lark presence are included in the SPAs Network, where specific management measures for bird conservation are expected.

Regarding habitat preferences, our results confirm that Dupont’s lark is a strong specialist, with a high dependency on sclerophyllous scrub. It seems to tolerate extensive cereal steppes (dry farming), and the probability of presence values increase slightly when up to 20% of the cell area is covered by this land category. This suggests the species' preference for steppe and dry farming mosaics, but the probability of presence decreases dramatically when dry farming is above 60%. The species' tolerance to more intensive types of farming is much less. As several records confirm the use of dry farming crops during winter accompanying other lark species [7, 26], our results suggest that Dupont’s lark would benefit from the proximity of these areas to the natural breeding steppe lands, where alternative resources can be found during unfavourable periods (such as food availability in summer or more temperate conditions in winter). Finally, habitat selection studies define slope as a critical factor for Dupont’s lark presence, situating the threshold between 10 and 15% [9]. Our results support these data, as we found a peak of probability of presence in flat terrains (around 5%), decreasing linearly to 15% and with a more marked reduction above this threshold (Fig 2).

NDVI was the main predictor of Dupont’s lark distribution accounting for most of the contribution in the final model (19.90%) and with an AUC value over 80 in the jackknife test, together with several habitat predictors (farming, forest and sclerophyllous scrub), which supports the strong dependence of the species on a very specific habitat type. The presence of forest cover and farming different from dry agriculture are negative for the species.

Climate has also shown great importance in Dupont’s lark probability of presence, as with other steppe bird species [40, 59]. This dependence on thermal conditions in a small bird species could compromise its distribution in the scenario of future climate change (see below).

The range contraction analysis also showed some alarming results. The reduction in the distribution from pre-2000 to post-2000 was of 44.06%. This tendency seems to be generalised across the whole Spanish distribution range, with the extinction of the smallest and most peripheral nuclei [13, 15, 16]. The extinction process was explained by both anthropic and climatic factors. The effect of decreases in population density on cell extinction, even in areas where the population density is among the lowest in Europe (in the year 2009, in the core area of the species, Soria province: 9.2 inhab./km^2^; Teruel province: 9.8 inhab./km^2^; Guadalajara province: 19.5 inhab./km^2^; in some areas, as SPA Páramos de Barahona, in Soria: 3.0 inhab/km^2^; National Institute of Statistics database) suggests a negative link between rural abandonment (and the loss of traditional extensive grazing) and species occupancy. Dupont’s lark has been associated with extensive grazing due to its effect on maintaining plant architecture and dung supplies, which in turn, provide food for this insectivorous species [9, 29, 60]; own unpublished data). In terms of climate, a higher temperature range and a reduction in annual precipitation were associated with a higher probability of extinction. Although at a global scale Dupont’s lark occupies areas with high temperature range and low annual precipitation (as these climatic conditions shape its habitat), at a local scale (1x1 km cell) the species seems to be favoured by less extreme conditions. In the case of temperature this may be related to thermoregulation needs, which are particularly important in a small organism with a high surface:volume ratio [40]. In the case of water availability, a decrease in aridity could provide more favourable local conditions in relation to productivity and food availability [38, 39]. This result suggests that, despite its tolerance for continental and dry climates, future scenarios of climate change could dramatically affect the species, as predicted trends in these variables suggest increases in temperature range [61] and aridity [62]. More research is needed on this topic.

Finally, the installation of wind farms in a buffer of 4 km may have a significant effect on the probability of extinction of the species. Despite the fact that this variable was excluded from the final averaged model, further research and specific studies are needed, as other works have indicated a negative effect on the species [32] and expert opinion considers this factor as one of the main threats to Dupont’s lark [9, 15, 29].

## Supporting information

S1 Table Variables correlation. Correlation matrix to identify those explanatory variables that shared over 75% of information. January and April temperatures, January and pre-breeding rainfalls and EVI index were removed to avoid high autocorrelations.
(DOCX)

S2 Table Selection of the bias correction method. AUC values for all possible models resulting from the combination of the two bias correction methods, to select those that accounted for the highest value (unbiased locations file with all surface of analysis, AUC = 0.926).
(DOCX)

S3 Table Selection of the land use categories that represent the adequate habitat. Distribution of the occurrence locations during the study period, in CORINE 2006 land cover categories. Those that accumulated 95% of the observations (categories 323, 321, 333, 243 and 211) were selected in CORINE 2012 and merged to create a layer that represents the current distribution of the habitat preferred by the species.
(DOCX)

S4 Table Special Protection Areas (SPAs) with presence of Dupont’s lark in Spain. Regions and provinces affected, ranked by the surface area occupied by the species.
(DOCX)

S5 Table Important Bird Areas (IBAs) with presence of Dupont’s lark in Spain. Regions and provinces affected, ranked by the surface area occupied by the species.
(DOCX)

S1 File Potential distribution of Dupont’s lark in Spain. Shapefile (*Chersophilus_duponti*.*shp*) showing the 1x1 km grid of the study area. 1 represents the cells with confirmed presence in the post-2000 period (n = 1370), 2 represents the cells with unconfirmed but potential presence (n = 5575). The rest are the cells with improbable presence (value of probability under the mean).
(ZIP)
