# Correction: Factors affecting Dupont's lark distribution and range regression in Spain

**DOI:** 10.1371/journal.pone.0219092

**Published:** 2019-06-24

**Authors:** Alexander García Antón, Vicente Garza, Jorge Hernández Justribó, Juan Traba

In Fig 4 caption, the color labels red and blue are incorrectly swapped. Please see the complete, correct Fig 4 caption here.

**Fig 4:**
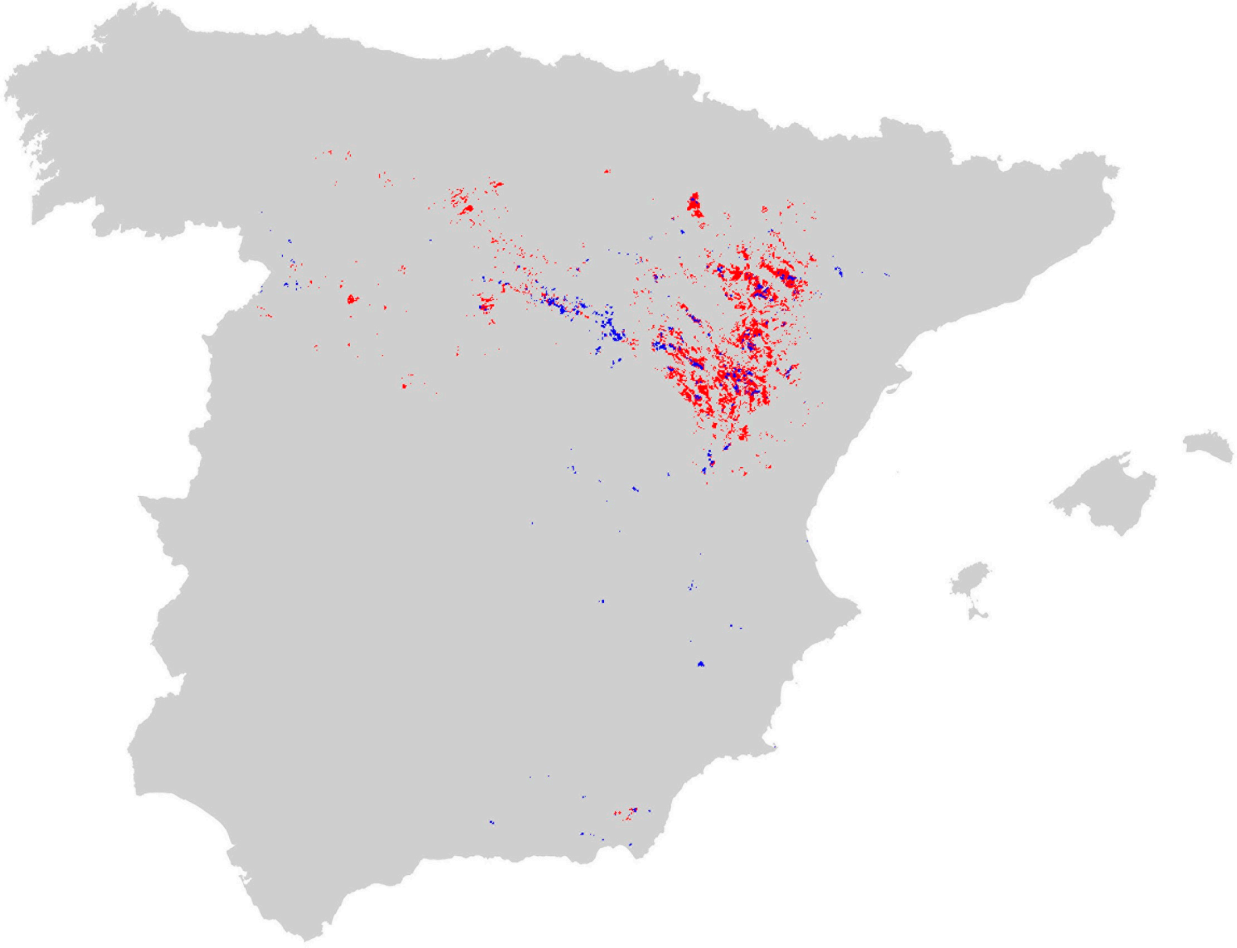
Map of potential distribution of Dupont’s lark in Spain. Grey indicates improbable presence (<0.49 probability threshold); red, potential but unconfirmed presence (>threshold, 5,575 cells); and blue, confirmed presence (1,370 cells). The map in SHP format is provided in Supporting Information (S1 File).
