# Supplementary material for: Factors affecting Dupont´s lark distribution and range regression in Spain
Source: PLoS One. 2019 Feb 15;14(2):e0211549. doi: 10.1371/journal.pone.0211549 (PMC6377091; doi:10.1371/journal.pone.0211549)
Supplement: S1 Table — Correlation matrix to identify those explanatory variables that shared over 75% of information. January and April temperatures, January and pre-breeding rainfalls and EVI index were removed to avoid high autocorrelations. (DOCX) [file pone.0211549.s001.docx]

**S1 Table. Variables correlation.**

|  | **Elevation** | **CV** | **Slope** | **January temp.** | **August temp.** | **April temp.** | **Temp. range** | **Annual prec.** | **January prec.** | **Prebreeding prec.** | **Artificial** | **Dry farming** | **Other farming** | **Farming +natural** |
| --- | --- | --- | --- | --- | --- | --- | --- | --- | --- | --- | --- | --- | --- | --- |
| **Elevation** | 1.00 |  |  |  |  |  |  |  |  |  |  |  |  |  |
| **CV** | -0.13 | 1.00 |  |  |  |  |  |  |  |  |  |  |  |  |
| **Slope** | 0.36 | 0.08 | 1.00 |  |  |  |  |  |  |  |  |  |  |  |
| **January temperature** | -0.89 | 0.13 | -0.25 | 1.00 |  |  |  |  |  |  |  |  |  |  |
| **August temperature** | -0.67 | 0.02 | -0.41 | 0.77 | 1.00 |  |  |  |  |  |  |  |  |  |
| **April temperature** | -0.87 | 0.11 | -0.38 | 0.88 | 0.88 | 1.00 |  |  |  |  |  |  |  |  |
| **Temperature range** | 0.22 | -0.14 | -0.28 | -0.19 | 0.46 | 0.14 | 1.00 |  |  |  |  |  |  |  |
| **Annual precipitation** | 0.16 | 0.07 | 0.44 | -0.13 | -0.59 | -0.39 | -0.67 | 1.00 |  |  |  |  |  |  |
| **January precipitation** | -0.06 | 0.08 | 0.27 | 0.16 | -0.25 | -0.07 | -0.51 | 0.87 | 1.00 |  |  |  |  |  |
| **Prebreeding precipitation** | -0.06 | 0.09 | 0.32 | 0.14 | -0.34 | -0.12 | -0.63 | 0.93 | 0.98 | 1.00 |  |  |  |  |
| **Artificial** | -0.16 | 0.05 | -0.11 | 0.15 | 0.09 | 0.13 | -0.09 | -0.03 | -0.03 | -0.01 | 1.00 |  |  |  |
| **Dry farming** | 0.00 | -0.07 | -0.40 | -0.09 | 0.06 | 0.01 | 0.21 | -0.25 | -0.23 | -0.25 | -0.06 | 1.00 |  |  |
| **Other farming** | -0.25 | 0.01 | -0.23 | 0.25 | 0.28 | 0.26 | 0.07 | -0.17 | -0.11 | -0.12 | -0.01 | -0.17 | 1.00 |  |
| **Farming+natural** | -0.03 | -0.01 | 0.00 | -0.01 | -0.07 | -0.03 | -0.11 | 0.05 | 0.03 | 0.04 | -0.03 | -0.10 | -0.10 | 1.00 |

|  | Farming +forest | Pasture | Scle. scrub | Other scrub | Scarce veget. | Unveget. | Wetland | Forest | NDVI | EVI | Transport Network 1 | Transport Network 2 | Population density | Cattle density |
| --- | --- | --- | --- | --- | --- | --- | --- | --- | --- | --- | --- | --- | --- | --- |
| **Farming+forest** | 1.00 |  |  |  |  |  |  |  |  |  |  |  |  |  |
| **Pasture** | -0.08 | 1.00 |  |  |  |  |  |  |  |  |  |  |  |  |
| **Sclerophyll scrub** | -0.14 | -0.05 | 1.00 |  |  |  |  |  |  |  |  |  |  |  |
| **Other scrub** | -0.14 | -0.07 | -0.08 | 1.00 |  |  |  |  |  |  |  |  |  |  |
| **Scarce vegetation** | -0.06 | -0.03 | -0.04 | -0.04 | 1.00 |  |  |  |  |  |  |  |  |  |
| **Unvegetated** | -0.04 | 0.02 | -0.02 | -0.02 | 0.03 | 1.00 |  |  |  |  |  |  |  |  |
| **Wetland** | -0.04 | -0.02 | -0.03 | -0.03 | -0.02 | 0.00 | 1.00 |  |  |  |  |  |  |  |
| **Forest** | -0.18 | -0.11 | -0.14 | -0.04 | -0.07 | -0.03 | -0.05 | 1.00 |  |  |  |  |  |  |
| **NDVI** | -0.01 | 0.05 | -0.02 | 0.26 | -0.14 | -0.08 | -0.10 | 0.54 | 1.00 |  |  |  |  |  |
| **EVI** | 0.08 | 0.09 | -0.13 | 0.12 | -0.17 | -0.09 | -0.13 | 0.31 | 0.88 | 1.00 |  |  |  |  |
| **Transport Network 1** | 0.02 | -0.02 | -0.05 | -0.07 | -0.02 | -0.01 | 0.00 | -0.06 | -0.06 | -0.01 | 1.00 |  |  |  |
| **Transport Network 2** | 0.09 | -0.05 | -0.11 | -0.10 | -0.05 | -0.05 | -0.04 | -0.07 | 0.05 | 0.15 | 0.14 | 1.00 |  |  |
| **Population density** | -0.02 | -0.02 | -0.04 | -0.04 | -0.01 | -0.01 | 0.03 | -0.04 | -0.05 | -0.04 | 0.16 | 0.10 | 1.00 |  |
| **Cattle density** | 0.09 | 0.07 | -0.01 | -0.07 | -0.03 | -0.02 | 0.00 | -0.09 | -0.08 | -0.03 | -0.02 | -0.03 | -0.04 | 1.00 |
