# Supplementary material for: Factors affecting Dupont´s lark distribution and range regression in Spain
Source: PLoS One. 2019 Feb 15;14(2):e0211549. doi: 10.1371/journal.pone.0211549 (PMC6377091; doi:10.1371/journal.pone.0211549)
Supplement: S2 Table — AUC values for all possible models resulting from the combination of the two bias correction methods, to select those that accounted for the highest value (unbiased locations file with all surface of analysis, AUC = 0.926). (DOCX) [file pone.0211549.s002.docx]

**S2 Table. Selection of the bias correction method.**

| **Backgr. extension** | **All (n=14,476)** | **Unbiased (n=1,370)** |
| --- | --- | --- |
| **10 km** | 0.793 | 0.876 |
| **25 km** | 0.708 | 0.909 |
| **50 km** | 0.710 | 0.915 |
| **100 km** | 0.702 | 0.922 |
| **All surface** | 0.706 | 0.926 |
