# Supplementary material for: Factors affecting Dupont´s lark distribution and range regression in Spain
Source: PLoS One. 2019 Feb 15;14(2):e0211549. doi: 10.1371/journal.pone.0211549 (PMC6377091; doi:10.1371/journal.pone.0211549)
Supplement: S3 Table — Distribution of the occurrence locations during the study period, in CORINE 2006 land cover categories. Those that accumulated 95% of the observations (categories 323, 321, 333, 243 and 211) were selected in CORINE 2012 and merged to create a layer that represents the current distribution of the habitat preferred by the species. (DOCX) [file pone.0211549.s003.docx]

**S3 Table.** **Selection of the land use categories that represent the adequate habitat.**

| **CORINE 2006** | **Nº obs.** | **Percentage of total** | **Cum. Percentage** |
| --- | --- | --- | --- |
| **323** | 8520 | 58,92 | 58,92 |
| **321** | 3441 | 23,80 | 82,72 |
| **333** | 822 | 5,68 | 88,40 |
| **243** | 771 | 5,33 | 93,73 |
| **211** | 697 | 4,82 | 98,55 |
| **311** | 106 | 0,73 | 99,29 |
| **324** | 32 | 0,22 | 99,51 |
| **334** | 26 | 0,18 | 99,69 |
| **242** | 14 | 0,10 | 99,79 |
| **312** | 9 | 0,06 | 99,85 |
| **222** | 7 | 0,05 | 99,90 |
| **411** | 7 | 0,05 | 99,94 |
| **133** | 2 | 0,01 | 99,96 |
| **131** | 1 | 0,01 | 99,97 |
| **212** | 1 | 0,01 | 99,97 |
| **221** | 1 | 0,01 | 99,98 |
| **223** | 1 | 0,01 | 99,99 |
| **313** | 1 | 0,01 | 99,99 |
| **331** | 1 | 0,01 | 100,00 |
| **Total** | 14460 | 100,00 |  |
