# Supplementary material for: Factors affecting Dupont´s lark distribution and range regression in Spain
Source: PLoS One. 2019 Feb 15;14(2):e0211549. doi: 10.1371/journal.pone.0211549 (PMC6377091; doi:10.1371/journal.pone.0211549)
Supplement: S4 Table — Regions and provinces affected, ranked by the surface area occupied by the species. (DOCX) [file pone.0211549.s004.docx]

**S4 Table. Important Bird Areas (IBAs) with presence of Dupont’s Lark in Spain.**

| Code | Name | Affected region/s | Affected province/s | IBA surface (km^2^) | Surf. with presence (km^2^) | % Surf. with presence |
| --- | --- | --- | --- | --- | --- | --- |
| 81 | Páramos de Layna y Medinaceli | Castilla y León,Castilla-La Mancha | Soria, Guadalajara | 296.82 | 118.70 | 39.99 |
| 79 | Tiermes - Caracena | Castilla y León,Castilla-La Mancha | Soria, Guadalajara | 597.78 | 112.23 | 18.77 |
| 80 | Altos de Barahona | Castilla y León,Castilla-La Mancha | Soria, Guadalajara | 499.40 | 87.61 | 17.54 |
| 189 | Paramera de Embid - Molina | Castilla-La Mancha, Aragón | Guadalajara, Zaragoza | 253.69 | 78.24 | 30.84 |
| 103 | Belchite - Mediana | Aragón | Zaragoza | 516.09 | 71.13 | 13.78 |
| 98 | Campo Visiedo | Aragón | Teruel | 332.17 | 67.23 | 20.24 |
| 435 | Muelas y Llanuras de Muniesa - Loscos - Anadón | Aragón | Teruel | 236.46 | 58.40 | 24.70 |
| 105 | Estepas de Monegrillo - Pina | Aragón | Zaragoza | 462.19 | 45.02 | 9.74 |
| 432 | Muelas y Parameras de Rillo - Pancrudo - Escucha | Aragón | Teruel | 189.76 | 44.84 | 23.63 |
| 96 | Parameras de Blancas | Aragón, Castilla-La Mancha | Teruel | 131.81 | 42.56 | 32.29 |
| 94 | Parameras del Río Jiloca | Aragón | Zaragoza, Teruel | 99.30 | 38.35 | 38.62 |
| 444 | Landete - Ademuz | Castilla-La Mancha, Comunidad Valenciana | Valencia, Cuenca | 100.38 | 36.83 | 36.69 |
| 441 | Altos de Alcolea del Pinar | Castilla-La Mancha, Castilla y León | Guadalajara, Soria | 88.34 | 25.84 | 29.25 |
| 436 | Parameras de Pozondón y Villar del Salz | Aragón | Teruel | 54.71 | 24.22 | 44.27 |
| 433 | Parameras de Alfambra | Aragón | Teruel | 159.42 | 22.62 | 14.19 |
| 431 | Llanura y Muelas de Valdejalón - Muel | Aragón | Zaragoza | 322.12 | 20.00 | 6.21 |
| 110 | Ballobar - Candasnos | Aragón | Huesca | 247.49 | 19.55 | 7.90 |
| 434 | Lomas de Ejulve y Molinos | Aragón | Teruel | 57.44 | 19.10 | 33.25 |
| 63 | Arribes del Duero - Fermoselle | Castilla y León | Zamora | 567.06 | 18.97 | 3.35 |
| 450 | Sierra del Picarcho | Región de Murcia | Murcia | 34.84 | 18.91 | 54.28 |
| 437 | Llanos de Riodeva y Cascante del Río | Aragón | Teruel | 32.41 | 15.52 | 47.89 |
| 188 | Alto Tajo y Tajuña | Castilla-La Mancha | Guadalajara | 1569.93 | 14.63 | 0.93 |
| 95 | Gallocanta | Aragón, Castilla-La Mancha | Zaragoza, Teruel | 286.24 | 12.45 | 4.35 |
| 53 | Cañón del Duratón | Castilla y León | Segovia | 117.50 | 12.38 | 10.54 |
| 440 | Llanuras del Alto Manubles | Aragón | Zaragoza | 54.96 | 12.28 | 22.34 |
| 90 | Las Bardenas Reales | Comunidad Foral de Navarra | Navarra | 584.06 | 12.00 | 2.05 |
| 215 | Sierra Alhamilla - Campo de Níjar - Sierra de Cabrera | Andalucía | Almería | 905.14 | 12.00 | 1.33 |
| 52 | Montejo de la Vega - Hoces del Riaza | Castilla y León | Segovia | 84.64 | 11.89 | 14.05 |
| 182 | Pétrola - Almansa - Yecla | Castilla-La Mancha, Región de Murcia | Albacete, Murcia | 851.70 | 11.61 | 1.36 |
| 106 | Los Monegros (Sur) | Aragón | Zagaroza, Huesca | 561.62 | 11.00 | 1.96 |
| 102 | Bajo Huerva | Aragón | Zaragoza | 421.86 | 10.61 | 2.52 |
| 439 | Llanos de Llumes | Aragón | Zaragoza | 36.06 | 10.54 | 29.23 |
| 438 | Altos del Norte de Teruel | Aragón | Teruel | 56.54 | 9.94 | 17.58 |
| 443 | Páramos de las Valeras y Hoz del Río Gritos | Castilla-La Mancha | Cuenca | 19.30 | 9.82 | 50.88 |
| 442 | Sierras de Uclés | Castilla-La Mancha | Cuenca | 38.42 | 8.71 | 22.67 |
| 447 | Parameras de Campo de Gómara | Castilla y León | Soria | 43.94 | 7.84 | 17.84 |
| 446 | Parameras de Soria | Castilla y León | Soria | 28.19 | 7.80 | 27.67 |
| 184 | Campo de Montiel | Castilla-La Mancha | Albacete | 1416.17 | 7.00 | 0.49 |
| 218 | Sierra de Gádor | Andalucía | Almería | 507.94 | 7.00 | 1.38 |
| 427 | El Temple - Lomas de Padul | Andalucía | Granada | 318.97 | 7.00 | 2.19 |
| 451 | Ablitas | Comunidad Foral de Navarra | Navarra | 7.66 | 6.33 | 82.64 |
| 100 | Cañones del Río Martín y Sierra de Arcos | Aragón | Teruel | 435.81 | 6.00 | 1.38 |
| 144 | Cogul - Alfés | Cataluña | Lérida | 228.25 | 5.68 | 2.49 |
| 428 | Estepas de Alcubierre | Aragón | Huesca | 25.97 | 4.89 | 18.83 |
| 430 | Lécera | Aragón | Zaragoza | 7.21 | 4.36 | 60.47 |
| 448 | Sierra de Cantadores - Losacio | Castilla y León | Zamora | 32.06 | 4.20 | 13.10 |
| 445 | Carboneras de Guadazaón - Los Poyales | Castilla-La Mancha | Cuenca | 29.60 | 4.00 | 13.51 |
| 429 | Llanos de Plasencia | Aragón | Zaragoza | 287.25 | 3.95 | 1.38 |
| 216 | Sierra y Salinas de Cabo de Gata | Andalucía | Almería | 467.00 | 3.92 | 0.84 |
| 114 | Campo de San Gregorio | Aragón | Zaragoza | 181.99 | 3.16 | 1.74 |
| 449 | Tábara | Castilla y León | Zamora | 6.16 | 2.58 | 41.88 |
| 44 | Páramos del Cerrato | Castilla y León | Palencia | 12.86 | 2.00 | 15.55 |
| 195 | Complejo Lagunar de Alcázar de San Juan - Quero | Castilla-La Mancha | Toledo | 663.12 | 2.00 | 0.30 |
| 192 | El Hito | Castilla-La Mancha | Cuenca | 214.08 | 1.99 | 0.93 |
| 214 | Hoya de Guadix | Andalucía | Granada | 691.09 | 1.83 | 0.26 |
| 113 | Sierra de Alcubierre | Aragón | Zaragoza | 653.40 | 1.11 | 0.17 |
| 101 | Saladas de Alcañiz | Aragón | Teruel | 66.07 | 1.00 | 1.51 |
| 185 | San Clemente - Villarrobledo | Castilla-La Mancha | Albacete | 1086.23 | 1.00 | 0.09 |
| 217 | Desierto de Tabernas | Andalucía | Almería | 263.93 | 1.00 | 0.38 |
| 169 | Mar Menor | Región de Murcia | Murcia | 153.43 | 0.99 | 0.65 |
| 191 | Embalses de Entrepeñas y Buendía | Castilla-La Mancha | Cuenca | 390.05 | 0.96 | 0.25 |
| 159 | Albufera de Valencia | Comunidad Valenciana | Valencia | 361.19 | 0.90 | 0.25 |
| 104 | Galachos y Riberas del Río Ebro | Aragón | Zaragoza | 60.09 | 0.84 | 1.40 |
| 149 | Puertos de Morella | Comunidad Valenciana | Castellón | 548.59 | 0.72 | 0.13 |
| 99 | Río Guadalope | Aragón | Teruel | 331.19 | 0.03 | 0.01 |
| 49 | Sierra de Alcarama y Río Alhama | La Rioja, Castilla y León | Soria | 120.61 | 0.01 | 0.01 |
| 92 | Sierra del Moncayo | Aragón, Castilla y León | Zaragoza | 498.10 | < 0.00 | < 0.00 |
