# Supplementary material for: Factors affecting Dupont´s lark distribution and range regression in Spain
Source: PLoS One. 2019 Feb 15;14(2):e0211549. doi: 10.1371/journal.pone.0211549 (PMC6377091; doi:10.1371/journal.pone.0211549)
Supplement: S5 Table — Regions and provinces affected, ranked by the surface area occupied by the species. (DOCX) [file pone.0211549.s005.docx]

**S5 Table. Special Protection Areas (SPAs) with presence of Dupont’s Lark in Spain.**

| Code | Name | Affected region/s | Affected province/s | IBA surface (km^2^) | Surf. with presence (km^2^) | % Surf. with presence |
| --- | --- | --- | --- | --- | --- | --- |
| ES0000302 | Parameras de Blancas | Aragón | Teruel | 40.33 | 70.77 | 70.77 |
| ES0000255 | Páramo de Layna | Castilla y León | Soria | 75.26 | 59.15 | 59.15 |
| ES0000160 | Hoz del río Gritos y páramos de Las Valeras | Castilla-La Mancha | Cuenca | 18.12 | 53.59 | 53.59 |
| ES0000305 | Parameras de Alfambra | Aragón | Teruel | 32.72 | 49.72 | 49.72 |
| ES0000308 | Parameras de Pozondón | Aragón | Teruel | 24.61 | 47.01 | 47.01 |
| ES4240023 | Lagunas y parameras del Señorío de Molina | Castilla-La Mancha | Guadalajara | 61.64 | 41.86 | 41.86 |
| ES0000448 | Hontanar - La Ferriza | Comunidad Valenciana | Valencia | 31.45 | 41.37 | 41.37 |
| ES0000304 | Parameras de Campo Visiedo | Aragón | Teruel | 177.72 | 21.57 | 21.57 |
| ES2430101 | Muelas del Jiloca: El Campo y La Torreta | Aragón | Zaragoza | 94.31 | 21.45 | 21.45 |
| ES0000136 | Estepas de Belchite - El Planerón - La Lomaza | Aragón | Zaragoza | 250.01 | 21.23 | 21.23 |
| ES0000203 | Altos de Barahona | Castilla y León | Soria | 428.81 | 20.68 | 20.68 |
| ES0000389 | Rentos de Orchova y vertientes del Turia | Castilla-La Mancha | Cuenca | 63.36 | 15.39 | 15.39 |
| ES0000180 | Estepas de Monegrillo y Pina | Aragón | Zaragoza | 245.33 | 15.35 | 15.35 |
| ES4160008 | Hoces del Río Riaza | Castilla y León | Segovia | 65.43 | 14.50 | 14.50 |
| ES0000115 | Hoces del Río Duratón | Castilla y León | Segovia | 50.42 | 13.41 | 13.41 |
| ES0000183 | El Basal, Las Menorcas y Llanos de Cardiel | Aragón | Huesca | 69.87 | 10.15 | 10.15 |
| ES0000021 | Secans de Mas de Melons-Alfés | Cataluña | Lérida | 76.17 | 7.38 | 7.38 |
| ES0000206 | Cañones del Duero | Castilla y León | Zamora | 175.46 | 7.10 | 7.10 |
| ES0000017 | Cuenca de Gallocanta | Aragón | Zaragoza | 152.21 | 4.91 | 4.91 |
| ES0000196 | Estepas de Yecla | Región de Murcia | Murcia | 42.44 | 4.52 | 4.52 |
| ES0000154 | Zona esteparia de El Bonillo | Castilla-La Mancha | Albacete | 172.80 | 4.05 | 4.05 |
| ES6110002 | Karst en yesos de Sorbas | Andalucía | Almería | 23.12 | 381 | 3.81 |
| ES0000094 | Parameras de Maranchón, hoz del Mesa y Aragoncillo | Castilla-La Mancha | Guadalajara | 463.01 | 3.35 | 3.35 |
| ES0000181 | La Retuerta y Saladas de Sástago | Aragón | Huesca, Zaragoza | 360.05 | 2.90 | 2.90 |
| ES0000360 | Cihuela-Deza | Castilla y León | Soria | 44.74 | 2.24 | 2.24 |
| ES0000303 | Desfiladeros del Río Martín | Aragón | Teruel | 447.86 | 1.46 | 1.46 |
| ES0000171 | El Plano-Blanca alta | Navarra | Navarra | 88.57 | 1.35 | 1.35 |
| ES0000091 | Humedales de La Mancha | Castilla-La Mancha | Toledo | 146.16 | 1.35 | 1.35 |
| ES0000300 | Río Huerva y Las Planas | Aragón | Zaragoza | 303.26 | 1.21 | 1.21 |
| ES0000306 | Río Guadalope - Maestrazgo | Aragón | Teruel | 542.44 | 0.78 | 0.78 |
| ES0000046 | Cabo de Gata - Níjar | Andalucía | Almería | 495.12 | 0.70 | 0.70 |
| ES0000260 | Mar Menor | Región de Murcia | Murcia | 145.26 | 0.65 | 0.65 |
| ES0000092 | Alto Tajo | Castilla-La Mancha | Guadalajara | 1912.54 | 0.48 | 0.48 |
| ES0000118 | Arribes del Duero | Castilla y León | Zamora | 1080.54 | 0.32 | 0.32 |
| ES0000163 | Sierra de Altomira | Castilla-La Mancha | Cuenca | 298.31 | 0.31 | 0.31 |
| ES0000471 | l'Albufera | Comunidad Valenciana | Valencia | 292.85 | 0.31 | 0.31 |
| ES0000295 | Sierra de Alcubierre | Aragón | Zaragoza | 421.08 | 0.24 | 0.24 |
| ES0000153 | Área esteparia del este de Albacete | Castilla-La Mancha | Albacete | 257.56 | 0.00 | 0.00 |
